# Increased MRI-based Brain Age in chronic migraine patients

**DOI:** 10.1186/s10194-023-01670-6

**Published:** 2023-10-06

**Authors:** Rafael Navarro-González, David García-Azorín, Ángel L. Guerrero-Peral, Álvaro Planchuelo-Gómez, Santiago Aja-Fernández, Rodrigo de Luis-García

**Affiliations:** 1 Laboratorio de Procesado de Imagen, Universidad de Valladolid, Valladolid, Spain; 2 Headache Unit, Department of Neurology, Hospital Clínico Universitario de Valladolid, Valladolid, Spain; 3 Department of Medicine, Universidad de Valladolid, Valladolid, Spain; 4 Cardiff University Brain Research Imaging Centre (CUBRIC), School of Psychology, Cardiff University, Cardiff, UK

**Keywords:** Neuroimaging, Biomarkers, Brain age, Migraine disorders, Machine learning

## Abstract

**Introduction:**

Neuroimaging has revealed that migraine is linked to alterations in both the structure and function of the brain. However, the relationship of these changes with aging has not been studied in detail. Here we employ the Brain Age framework to analyze migraine, by building a machine-learning model that predicts age from neuroimaging data. We hypothesize that migraine patients will exhibit an increased Brain Age Gap (the difference between the predicted age and the chronological age) compared to healthy participants.

**Methods:**

We trained a machine learning model to predict Brain Age from 2,771 T1-weighted magnetic resonance imaging scans of healthy subjects. The processing pipeline included the automatic segmentation of the images, the extraction of 1,479 imaging features (both morphological and intensity-based), harmonization, feature selection and training inside a 10-fold cross-validation scheme. Separate models based only on morphological and intensity features were also trained, and all the Brain Age models were later applied to a discovery cohort composed of 247 subjects, divided into healthy controls (HC, n=82), episodic migraine (EM, n=91), and chronic migraine patients (CM, n=74).

**Results:**

CM patients showed an increased Brain Age Gap compared to HC (4.16 vs -0.56 years, P=0.01). A smaller Brain Age Gap was found for EM patients, not reaching statistical significance (1.21 vs -0.56 years, P=0.19). No associations were found between the Brain Age Gap and headache or migraine frequency, or duration of the disease. Brain imaging features that have previously been associated with migraine were among the main drivers of the differences in the predicted age. Also, the separate analysis using only morphological or intensity-based features revealed different patterns in the Brain Age biomarker in patients with migraine.

**Conclusion:**

The brain-predicted age has shown to be a sensitive biomarker of CM patients and can help reveal distinct aging patterns in migraine.

**Supplementary Information:**

The online version contains supplementary material available at 10.1186/s10194-023-01670-6.

## Introduction

Migraine is a prevalent and chronic condition known for its recurrent and debilitating headache episodes. Migraine can be classified into two categories based on the frequency of headache days per month, namely episodic migraine (EM) and chronic migraine (CM) [1]. Due to the inherent characteristics of migraine and its widespread occurrence, it imposes a substantial burden on both individuals and society as a whole [2].

Migraine is associated with changes in the brain. Beyond the direct effects (i.e., the experience of pain during the ictal phase), neuroimaging studies have discovered alterations in the migrainous brain during the interictal phase encompassing both the structural and functional levels [3–5]. Differences between EM and CM have also been reported [6–8]. Even though the structure and function of the brain are also impacted by changes due to brain development and aging, the interplay between those and changes related to migraine has not been explored in depth. Bell et al. [9], for instance, focused on the pediatric age range, finding age- and puberty-dependent alterations in the functional connectivity of multiple networks in children with migraine using resting-state functional Magnetic Resonance Imaging (fMRI) and showing that brain changes associated with migraine begin in infancy and are modulated by development. Chong et al. [10] studied morphological changes of EM patients along age and found that patients with migraine have age-related thinning of regions compared to the control group. Using fluorodeoxyglucose positron emission tomography (FGD-PET), M. Lisicki et al. [11] showed that episodic migraine patients exhibit specific metabolic brain modifications while aging.

Recently, the so-called Brain Age paradigm has been proposed to explore the relationship between aging and disease [12]. Using machine learning techniques from neuroimaging data, chronological age can be accurately predicted in healthy individuals. After training a Brain Age model, the difference between an individual’s chronological age and the age predicted by the Brain Age model is usually referred to as “Brain Age Gap”, “Brain Age Gap Estimate” or “brain-predicted age difference” (brain-PAD), and has been proposed as an age-adjusted index of structural brain health. Research has shown the Brain Age paradigm to be sensitive to many neurological, psychiatric, and metabolic disorders, showing a positive Brain Age Gap, higher age compared- to the healthy brain, in disorders such as Alzheimer’s, schizophrenia, and type II diabetes, among others [13–15]. Conversely, protective sociological and lifestyle factors including years of education, physical exercise, playing music, or meditation have been associated with a negative Brain Age Gap [16–18]. An increased predicted Brain Age has even been associated with higher allostatic load and elevated overall mortality risk [19].

Even though other pain-related conditions have been studied using the Brain Age paradigm [20–23], to the best of our knowledge, migraine has not been explored from this perspective.

In this work, the Brain Age framework was employed to investigate migraine on a dataset composed of structural T1-weighted (T1w) MRI from EM and CM patients, together with normal controls. We hypothesized that migraine patients will exhibit an increased Brain Age Gap compared to healthy participants. Furthermore, we aimed to detect possible associations between the Brain Age Gap and clinical characteristics in the patient groups exploring the role of different imaging features.

## Materials and methods

Developing a robust Brain Age model involves several crucial steps. Firstly, it is imperative to assemble a diverse, broad, and representative dataset that encompasses neuroimaging data alongside corresponding chronological ages. The size of the dataset plays a significant role, as a larger dataset enables greater precision and generalizability in the final model. Subsequently, feature extraction is performed to capture pertinent information from the neuroimaging data. This process ensures that only informative and discriminative features are included in the model.

Once the features have been extracted, an appropriate machine-learning algorithm is selected for age prediction based on the neuroimaging data. Common choices include support vector machines or neural networks. The chosen algorithm is then trained using the dataset, and techniques such as regularization, cross-validation, and hyperparameter tuning are employed to optimize performance and prevent overfitting. The trained model is next evaluated using a separate dataset, employing metrics such as mean absolute error (MAE) or correlation coefficients to assess accuracy and generalization capabilities. This evaluation step provides valuable insights into the model’s performance and its ability to accurately estimate Brain Age.

Finally, the trained Brain Age model can be applied to new and unseen neuroimaging data. In our case, we apply it to a dataset composed of healthy controls, patients with episodic migraine, and patients with chronic migraine.

### Brain age model

To create and evaluate our age prediction models, we compiled a dataset (hereinafter referred to as *Model Creation Dataset*) consisting of 2,771 structural T1w MRI scans from different studies and databases that were publicly available. These include: the Dallas Lifespan Brain Study (DLBS) [24]; the Consortium for Reliability and Reproducibility dataset (CoRR) [25]; the Neurocognitive aging data release (NeuroCog) [26]; The OASIS-1 dataset [27]; the Southwest University Adult Lifespan Dataset (SALD) [28]; the Information eXtraction from Images dataset (IXI) [29]; and the CamCAN repository (available at http://www.mrc-cbu.cam.ac.uk/datasets/camcan/) [30, 31]. In addition to these, we included a set of healthy adults from the *Laboratorio de Procesado de Imagen* (LPI), our own institution. We selected only participants in good health and within the age range of 18 to 60 years. Individuals who presented neurological or psychological diagnoses or cognitive impairments were eliminated from the OASIS-1 and CoRR databases. Table 1 depicts the basic features of the *Model Creation Dataset*. Supplementary file 2 offers a detailed description of the included acquisitions for each database.

**Table 1:** Summary characteristics of the datasets used in the *Model Creation Dataset*, sorted by median age

Dataset	No. Cases	No. Females (%)	Age Range (Median)
*CoRR*	935	479 (51.2)	18-60 (22)
*NeuroCog*	190	107 (56.3)	18-60 (22)
*LPI*	91	33 (36.3)	18-53 (24)
*OASIS-1*	218	126 (57.8)	18-60 (25)
*IXI*	384	203 (52.9)	20-60 (39)
*DLBS*	174	112 (64.4)	21-60 (39)
*SALD*	393	249 (63.3)	19-60 (40)
*CamCan*	386	198 (51.2)	18-60 (42)
*Model Creation Dataset*	2771	1507 (54.4)	18-60 (28)

From the T1w images, FastSurfer [32] was employed to extract a total of 1,479 features. Fastsurfer uses Deep Learning to perform brain segmentation based on the Desikan-Killiany atlas [33, 34]. Two types of features were extracted:

- 624 morphological features, including whole brain features, the volume of cortical and subcortical gray matter regions and white matter regions from the atlas, as well as the surface, thickness and curvature of the cortical regions. This feature set will be referred to as *Morphological Feature Set*.
- 855 intensity-based features extracted from the same regions. This feature set will be referred to as *Intensity Feature Set*.

Together, all 1,479 features make up the *Combined Feature Set*. The three feature sets obtained using this procedure were the basis for further analysis.

To ensure their quality, segmentations were manually inspected. In Supplementary Table 1, Supplementary Fig. 1 and Supplementary Table 2, features and regions of interest are covered in greater detail.

MRI acquisitions obtained at different sites and/or using different protocols can differ in their intensity levels, which can introduce a bias in the Brain Age-predicting models. In order to cope with this problem, we used ComBat [35, 36] to harmonize the features from the *Intensity Feature Set* and the *Combined Feature Set*, using age, sex, and estimated total intracranial volume (eTIV) as covariates.

Afterwards, the cases were randomly divided into an 8:1:1 ratio for training, validation, and testing. We conducted a 10-fold cross-validation training procedure over the harmonized features to predict age. We flattened outliers of each feature, defined as values on the 97.5th or 2.5th percentile. In addition, each characteristic was adjusted to the range (-1, 1) using min-max normalization. Each fold underwent feature selection, defining three sets of 20, 30, and 40 characteristics. The selection of features was accomplished in two steps. Initially, a filter was used to choose the first decile features based on the mutual information between features and age in the training set. Next, the final features were chosen by employing a forward feature selection approach with gaussian mixture models to optimize the mutual information between a subset of features and age [37].

**Fig. 1:**
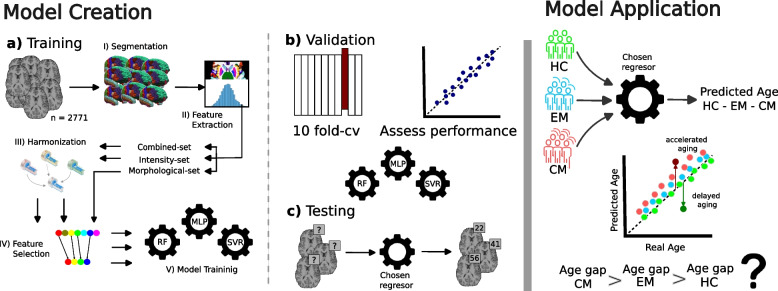
Comprehensive illustration of the methodologies employed for the training of the Brain Age models and the generation of brain-predicted ages. *Model Creation* shows the steps taken to train the Brain Age model on the *Model Creation Dataset* and choose the final model applied on the *Application Dataset*: **a** Image processing includes Fastsurfer for brain segmentation and extraction of intensity and morphological features, thus building three feature sets: the *Morphological Feature Set*, *Intensity Feature Set* and the *Combined Feature Set*. For each of these feature sets, a feature selection procedure is performed in a 10-fold cross-validation scheme creating feature sets of 20, 30 and 40 features to feed the machine learning models (SVR, RF and MLP) for each fold. **b** Validation is performed to select the best combination of feature set size and machine learning technique. **c** Test on the *Model Creation Dataset* to assess the performance of the Brain Age prediction model. *Model Application* depicts the use of the chosen model on the patient and healthy groups. Brain Age Gap is calculated as the difference between the predicted and the actual age. Differences in Brain Age Gap are then analyzed

As regressors, support vector regressor (SVR), random forest (RF), and a multilayer perceptron (MLP) were evaluated. Figure 1 depicts the process followed. By combining these three regressors with distinct feature sets of 20, 30, and 40 characteristics for each fold, a total of 90 models were trained.

Predictions were obtained for the validation and test set for each fold. Validation results were used to select the Brain Age model to be selected as the best-performing, while test results were exclusively employed to report the accuracy of the Brain Age model on the *Model Creation Dataset*.

We are aware that Brain Age models suffer from regression dilution, which causes bias in Brain Age predictions. Therefore, in order to avoid possible spurious associations, a correction for this effect was applied [38, 39]. A linear regression was fitted between the real age and validation results of each of the regressors of the ensemble. The intercept $(\alpha )$ and slope $(\beta )$ of each fit were then used to correct the predictions obtained for the studied groups following the equation:

1 $$\begin{aligned} \text {CorrectedPredictedAge} = (\text {Predicted Age} - \beta ) / \alpha \end{aligned}$$

This approach was repeated for each of the aforementioned feature sets. The training procedure was performed using the scikit-learn Python library for machine learning [40]. The SVR and RF models were imported from the library while the MLP was implemented using PyTorch [41]. Details of the MLP implementation and the hyperparameters for each model are described in Supplementary Table 3.

### Participants

A total of 391 subjects were included in this study, on which the Brain Age model previously described was applied. First, we employed healthy subjects from the Nathan Kline Institute - Rockland Sample (NKI-RS) dataset [42], for the purpose of external validation (n = 144). Next, and in order to study the influence of migraine on Brain Age, we employed a dataset composed of healthy controls (HC, n=82), EM (n=91), and CM patients (n=74). This dataset will be hereinafter referred to as *Application Dataset*.

Patients were recruited from the outpatient headache unit at the Hospital Clínico Universitario de Valladolid (Spain), a public tertiary care institution that accepts patients from both secondary care and primary care. Inclusion criteria were: a) migraine diagnosis using the third edition of the International Classification of Headache Disorders (ICHD-3) beta and ICHD-3 criteria [1, 43]; b) a stable clinical state in the last six months; and c) expressed willingness to partake in the study, coupled with the voluntary signing of the informed consent document. We excluded patients with the following conditions: a) high-frequency episodic migraine, with 10 to 14 headache days per month; b) other painful conditions; c) known major psychiatric diseases (described as anamnesis or the presence of depression or anxiety in the Hospital Anxiety and Depression Scale [44]); d) other neurological diseases; e) drug or substance abuse; and f) pregnancy. At the time of inclusion, no preventive treatment was given to the patients. Participants were requested to complete a headache diary and were diagnosed with EM if they experienced 10 headache days per month or less and CM if they met the ICHD-3 criteria.

Age- and sex-matched HC were recruited through hospital and university colleagues, as well as ads at these institutions, using convenience sampling and snowball sampling. No HC were included if they had a current or previous history of migraine, or if they had any other neurological or mental disorder following the same exclusion criteria as for migraine patients.

We gathered sociodemographic and clinical data from all patients, including migraine illness duration (years) and headache and migraine frequency (days per month).

The study was approved by Hospital Clínico Universitario de Valladolid’s local Ethics Committee (PI: 14- 197). All participants read and signed a written consent form before their participation.

### Image acquisition and processing

High-resolution 3D T1w MRI data were acquired for all subjects using a Philips Achieva 3T MRI unit (Philips Healthcare, Best, the Netherlands) with a 32-channel head coil in the MRI facility at the Universidad de Valladolid (Spain). Acquisition parameters were the following: Turbo Field Echo (TFE) sequence, repetition time (TR) = 8.1 ms, echo time (TE) = 3.7 ms, flip angle=$8^o$, $256\times 256$ matrix size, $1\times 1\times 1$ mm$^3$ of spatial resolution, and 160 sagittal slices covering the whole brain. Image acquisitions for migraine patients were performed during interictal periods (defined as at least 24 hours from the last migraine attack). Details about the acquisition protocols of each public dataset are described in Supplementary Table 4. If more information is required, further details can be found in each portal of the databases used.

Following the image acquisition, image segmentation, feature extraction and harmonization were also performed on the *Application Dataset* as described for the creation of the Brain Age model. Next, Brain Age was estimated for each participant, including correction from the regression dilution. Since we conducted a 10-fold cross-validation for the training, validation and testing of the Brain Age model, an ensemble formed with the average result of the trained model from each fold was used to obtain the final prediction. Finally, the Brain Age Gap was calculated as the difference between the corrected predicted age and the chronological age of each individual.

### Model interpretation

The significance of each imaging feature in the Brain Age estimation was evaluated using SHapley Additive exPlanations (SHAP) [45]. SHAP is a game-theory-based model-agnostic explanation method for machine learning models that evaluates the contribution of each feature to a given prediction. By employing this approach, a group-level comparison of distinct brain imaging features can be conducted to determine their significant contribution to age prediction. Additionally, the evaluation of the influence of individual features on each participant’s Brain Age prediction is made possible, as exemplified in the study conducted by Ballester et al. [46].

The SHAP value for a particular feature for a specific prediction can be interpreted as the difference in the prediction when that feature is omitted from the model. SHAP values reinterpret complex models as a linear function:

2 $$\begin{aligned} g(z') = \phi _0 + \sum \phi _i z'_i \end{aligned}$$

where *z’* is a simplified version of the input features of the model, $\phi _0$ is a reference value of the model (in our case is a value close to the average age of the training data), and $\phi _i$, the attribute effect of the feature which deviates the prediction from the reference value. In a database with N participants and M features, for example, SHAP generates an $N\times M$ matrix, where each value represents the contribution of feature *m* to the prediction of participant *n*.

We calculated the SHAP value for each subject for a deeper understanding of the regressors. Since many features are repeated across the different regressors, we summed up the contribution of repeated features into a single value. The final matrix was divided by 10 since our ensemble model is the average of the results of the 10 regressors trained during the 10-fold cross-validation.

Once we had the final matrix, we aggregated the values for each of the groups considered (HC, EM and CM) by summing up the absolute values of the matrix along the participant’s axis. The best 15 features in terms of their absolute contribution for each group were selected for each model of the ensemble. Unique features among the three groups studied were selected as the most informative features.

### Statistical analysis

The performance evaluation of the Brain Age models was conducted using two metrics: the MAE and Pearson’s correlation coefficient (*r*). The MAE was calculated as the average of the absolute values of the residuals, which were obtained by subtracting the predicted age from the actual age for each individual in the group. The MAE serves as a comprehensive measure of the prediction error across the entire group, with lower values indicating a better fit. On the other hand, Pearson’s correlation coefficient measures the strength and direction of the linear relationship between the predicted ages and the real ages. Higher values of *r* indicate a better fit of the model. The specific formulas for these metrics can be found in equations (3) and (4). Further exploration of these performance metrics can be found in the work from de Lange et al. [39].

3 $$\begin{aligned} MAE = \frac{1}{N}\sum _{i=1}^{N}|\tilde{y}_i-y_i| \end{aligned}$$

4 $$\begin{aligned} r = \frac{ \sum (y_i-\bar{y})(\hat{y}_i-\bar{\hat{y}})}{\sqrt{\sum (y_i-\bar{y})^2\sum (\hat{y}_i-\bar{\hat{y}})^2}} \end{aligned}$$

We assessed the normality and homogeneity of variance for age and duration of migraine in the *Application Dataset* using the Kolmogorov-Smirnov test and Levene’s test for equality of variances, respectively. If the null hypothesis was not rejected in both tests, we performed a one-way analysis of variance (ANOVA) to determine significant differences in the ages of the three groups. Gender-significant differences were identified using a chi-square test. For comparing clinical characteristics between migraine patients (i.e., duration of migraine history in years for both groups of patients), we used a two-tailed unpaired t-test if the null hypothesis was not rejected by the Kolmogorov-Smirnov; alternatively, we used the Mann-Whitney U test.

An Analysis of Covariance (ANCOVA) was conducted on the Brain Age Gap outcomes across the three groups, incorporating eTIV and sex as covariates. Upon ascertaining that the *p*-value suggested a need for further investigation, pairwise comparisons between the groups were carried out, keeping the same covariates. The analysis was made using all subjects followed by sex-specific comparisons. To verify that the Brain Age Gap calculated for each group was approximately normal and that the variances between groups were comparable, we performed the Kolmogorov-Smirnov test and the Levene test. In the case of a negative Levene’s test, we verified that the variance ratio did not exceed 2 [47]. We reset the *P* value threshold correcting for multiple comparisons using the Bonferroni correction method (P threshold = 0.0167). To conduct a more detailed examination of the variations among the groups, we computed the Cohen’s d statistic.

Regarding the model interpretation, We conducted a Kruskal-Wallis test on the SHAP values obtained for each of the highly important features of each regressor trained to analyze differences in feature importance among the studied groups. A non-parametric test was chosen due to the non-normality of the SHAP values. To account for multiple comparisons, we applied the Benjamini-Hochberg correction method. We performed pairwise comparisons using the post-hoc Connover-Iman test, correcting its *p*-values for multiple comparisons using the Benjamini-Hochberg method if the Kruskal-Wallis Test was significant.

To deepen our understanding of how migraines influence brain health, we examined the role of brain volume in linking the frequency of headaches in migraine patients to the Brain Age Gap. This type of analysis, known as mediation analysis, is a standard method in the realm of neuroimaging [48]. Utilizing a single-tier, three-variable mediation model, we sought to discover if segmented brain volume could serve as a mediator (M) between headache frequency (independent variable, X) and the Brain Age Gap (dependent variable, Y). We adjusted for potential confounding factors like age and sex in the model. To assess the validity of the mediation effect, we employed a bias-corrected bootstrap technique, using 10,000 random samples. For a more nuanced understanding, we looked at these relationships both across the general population of migraine patients and within individual subgroups.

Finally, we computed the Pearson’s correlation coefficient to assess the association between the Brain Age Gap and the clinical characteristics of the migraine groups. Corrections for multiple comparisons were made using the Benjamini-Hochberg method. We also explored the relationship between the imaging features that were selected as highly important during the SHAP analysis and these clinical characteristics, making additional adjustments for multiple comparisons using the Benjamini-Hochberg approach. Partial correlation analyses were performed to control for age as a confounding variable given its potential influence on the duration of migraine and chronic migraine. All statistical procedures were executed in Python.

## Results

### Demographics

**Fig. 2:**
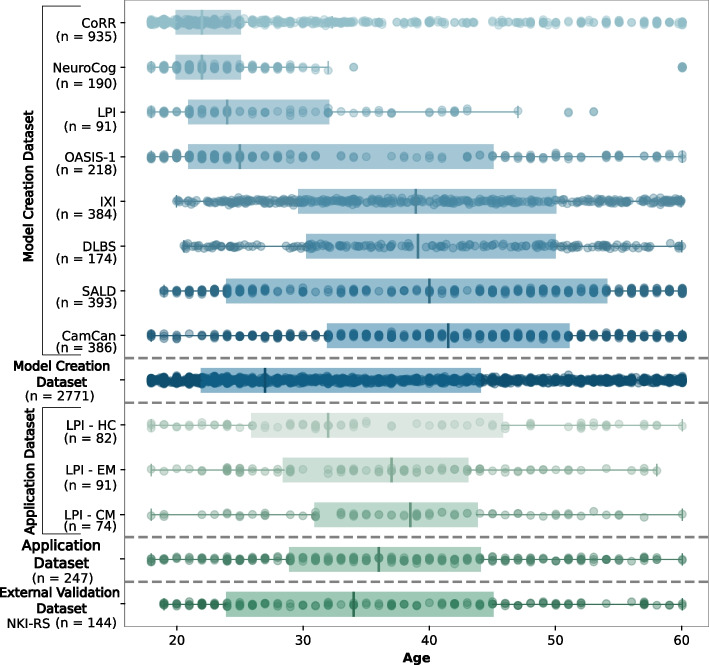
Age distributions of studies in the *Model Creation Dataset* and the *Application Dataset*, ordered by median age

There were no significant differences between the groups (HC, EM and CM) in the *Application Dataset* regarding age or sex. Table 2 shows the demographic and clinical characteristics of the dataset, while Fig. 2 shows the age distribution of the subjects, together with those in the *Model Creation Dataset*.

**Table 2:** Demographic and clinical characteristics for the *Application Dataset* and the *External Validation Dataset*. Not all patients completed the headache diary. Complete data was available from 87 EM patients and 72 CM patients

	NKI-RS (n=144)	HC (n=82)	EM (n=91)	CM (n=74)	Statistical Test
*Woman* $N^{\underline{\circ }}$. *(%)*	89 (61.8 %)	68 (82.9 %)	75 (82.4 %)	68 (91.9 %)	sex - $\chi ^2$ = 3.56, P = 0.17^a^
*Age, y*	35.5 ± 12.0	35.7 ± 12.0	36.4 ± 9.9	37.8 ± 9.7	age - ANOVA = 0.86, P = 0.43^b^
EM n = 87; CM n = 72;					
*Duration of migraine history, y*			14.1 ± 10.7	19.8 ± 10.7	t = -3.32, P = 0.001^c^
*Duration of Chronic migraine, mo*				28.4 ± 35.3	
*Headache frequency d/mo*			3.5 ± 2.1	23.5 ± 6.0	U = 66, P < 0.001^d^
*Migraine frequency d/mo*			3.6 ± 2.0	13.6 ± 6.8	U = 225.5, P < 0.001^d^

Data expressed as mean ± SD

^a^Chi-square test

^b^ANOVA

^c^Two-tailed, unpaired Student *t* test

^d^Mann-Whitney U test

### Performance of the Brain Age model

Table 3 provides a summary of the validation results for the models tested with the three feature sets studied after age bias correction. Results before the bias correction can be found in Supplementary Table 5. The ensemble model formed by MLPs with a set of 40 selected features provided the greatest performance among the evaluated models in all feature sets and was therefore selected to perform all the Brain Age predictions whose results are described next.

**Table 3:** Validation results for the three regressors tested. Results are given as the average and the standard deviation of the values obtained from each fold of the 10-fold cross-validation scheme after age bias correction. The values in bold show the combination with the best result

		SVR		RF		MLP	
		MAE	r	MAE	r	MAE	r
**Combined Feature Set**	**20 features**	7.40 ± 0.50	0.82 ± 0.02	6.59 ± 0.51	0.84 ± 0.02	5.99 ± 0.55	0.86 ± 0.02
	**30 features**	7.21 ± 0.50	0.83 ± 0.02	6.59 ± 0.48	0.84 ± 0.02	5.81 ± 0.47	0.86 ± 0.02
	**40 features**	7.10 ± 0.42	0.83 ± 0.02	6.58 ± 0.48	0.84 ± 0.02	**5.79** $\varvec{\pm }$ **0.38**	**0.87** $\varvec{\pm }$ **0.01**
**Morphological Feature Set**	**20 features**	8.49 ± 0.66	0.78 ± 0.02	8.75 ± 1.05	0.76 ± 0.03	7.46 ± 0.85	0.80 ± 0.02
	**30 features**	8.18 ± 0.65	0.79 ± 0.02	8.62 ± 0.93	0.77 ± 0.03	7.31 ± 0.76	0.81 ± 0.02
	**40 features**	7.96 ± 0.55	0.80 ± 0.02	8.50 ± 0.74	0.77 ± 0.02	**7.13** $\varvec{\pm }$ **0.52**	**0.81** $\varvec{\pm }$ **0.02**
**Intensity Feature Set**	**20 features**	9.25 ± 0.67	0.75 ± 0.02	9.12 ± 0.68	0.75 ± 0.02	8.39 ± 0.76	0.77 ± 0.02
	**30 features**	9.15 ± 0.75	0.75 ± 0.02	9.10 ± 0.75	0.76 ± 0.02	8.33 ± 0.93	0.78 ± 0.03
	**40 features**	9.09 ± 0.65	0.76 ± 0.02	9.19 ± 0.77	0.75 ± 0.02	**8.24** $\varvec{\pm }$ **0.68**	**0.78** $\varvec{\pm }$ **0.02**

For the test data, training on the *Model Creation Dataset*, the Brain Age model working with the *Combined Feature Set* obtained an MAE and r of 5.95 years and 0.85. On the *External Validation Dataset* (NKI-RS) [42], this same model yielded an MAE and Pearson’s correlation of 6.19 years and 0.83, respectively. For the *Application Dataset*, we obtained a MAE and r of 6.26 years and 0.84 (HC group). With regard to the Brain Age model working only with the *Morphological Feature Set*, its performance was MAE = 7.13 years and r = 0.80 on the *Model Creation Dataset*. This model obtained an MAE = 6.92 years and r = 0.82 on the NKI-RS dataset and MAE = 7.83 years and r = 0.74 on the HC group of the *Application Dataset*. Finally, the Brain Age model operating solely with the *Intensity Feature Set* yielded MAE = 8.19 years and r = 0.78 on the *Model Creation Dataset*, an MAE = 8.63 years and r = 0.73 on the NKI-RS dataset and MAE = 9.27 and years r = 0.64 for the HC group of the *Application Dataset*. Results on the NKI-RS dataset are further detailed at Supplementary Fig. 2.

### Brain Age Gap in migraine

**Fig. 3:**
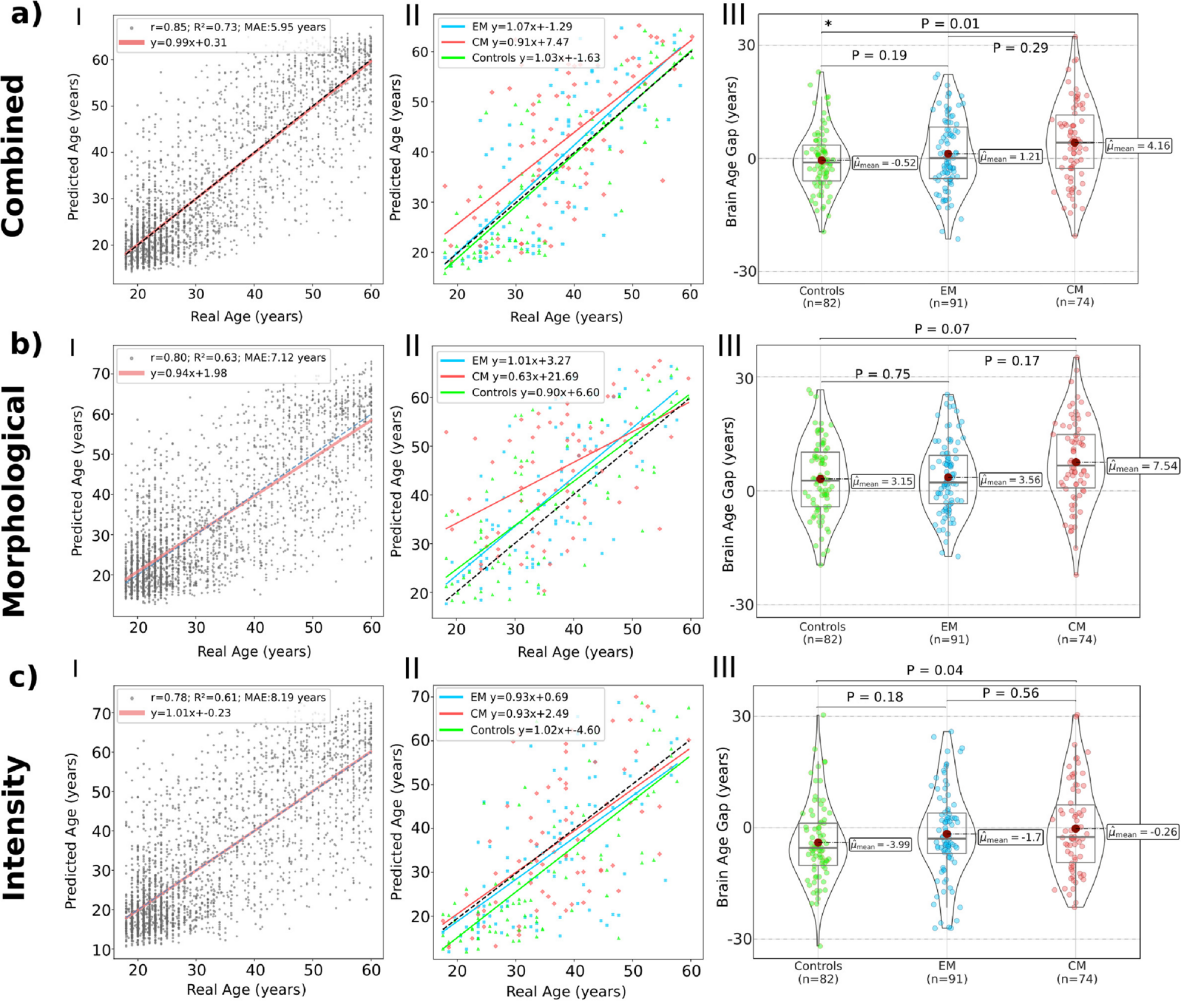
The results of each of the regressors build are shown. The MLPs selecting 40 features demonstrated the best results in validation for all the feature sets. For each trained regressor on every feature set: I) Ensemble MLPs result in the test set of each fold of *Model Creation Dataset*. II) Distribution of the Brain Age Gap values obtained for each of the studied groups. III) Brain Age Gap for the three groups. Statistical significance is denoted by an asterisk (*) to indicate significant findings

The outcome of the ANCOVA featuring three levels yielded a *p*-value of 0.053, suggesting potential significant differences in the pairwise comparisons. Using the *Combined Feature Set*, CM patients exhibited a statistically significant increased Brain Age Gap (average +4.16 vs -0.52 years, P = 0.010) compared to HC. EM patients showed an intermediate Brain Age Gap (average +1.21 years), and neither comparisons with HC nor CM yielded statistical significance. We computed Cohen’s d for the Brain Age Gap outcome of the combined regressor and obtained the following results. For the EM vs HC groups, it yielded 0.19; for CM vs HC, the value was 0.49; and for CM vs EM, the result was 0.29. This shows a moderate difference between the Brain Age Gap of CM vs HC. Figure 3 graphically depicts these results, together with scatterplots showing the brain-predicted age and the chronological age in both the *Model Creation Dataset* and the *Application Dataset*. Details of the ANCOVA results for each regressor can be found at Supplementary Tables 6, 7 and 8.

When only employing the *Morphological Feature Set*, CM showed an increased Brain Age Gap with respect to both HC and EM (+7.54 vs 3.15 and 3.56 years, respectively), although these differences were not statistically significant (P = 0.07 and P = 0.17). Interestingly, the average Brain Age Gap for HC and EM were very similar in this case. Cohen’s d yielded a small difference between the EM and HC groups (0.04) and a modarate difference between the CM and HC groups (0.42) and the CM and EM group (0.39).

Finally, when only employing the *Intensity Feature Set*, CM again showed an increased Brain Age Gap with respect to HC and EM, although differences were not statistically significant in this case either. Cohen’s d yielded 0.21 between EM and HC, 0.33 between CM and HC and 0.12 between the CM and EM group. Figure 3 and Table 4 depict these results.

**Table 4:** ANCOVA results for Brain Age Gap calculated for each regressor trained. Normality and equality of variances were tested before applying the ANCOVA. Sex and eTIV were included as covariates. The $\eta _p^2$ effect sizes were small ($\eta _p^2 < 0.06$) for all comparisons. Statistically significant elements are shown in bold

		ANCOVA HC-EM	ANCOVA HC-CM	ANCOVA EM-CM
**Combined Feature Set**	***F*-value**	1.734	6.796	1.110
	**Effect size (**$\varvec{\eta }_{\varvec{p}}^{\varvec{2}}$**)**	0.012	0.043	0.007
	***p*-value**	0.190	**0.010**	0.294
**Morphological Feature Set**	***F*-value**	0.102	3.237	1.924
	**Effect size (**$\varvec{\eta }_{\varvec{p}}^{\varvec{2}}$**)**	< 0.001	0.021	0.012
	***p*-value**	0.750	0.074	0.167
**Intensity Feature Set**	***F*-value**	1.802	4.094	0.336
	**Effect size (**$\varvec{\eta }_{\varvec{p}}^{\varvec{2}}$**)**	0.011	0.026	0.002
	***p*-value**	0.181	0.045	0.563

The separate analysis for female subjects suggests that the trends found for the whole cohort are maintained for the female group. No conclusions should be drawn, however, for the analysis of the male subjects given the small size of that subsample. Results of these analyses are provided in Supplementary Tables 9, 10 and Supplementary Fig. 3.

### Model interpretation

**Fig. 4:**
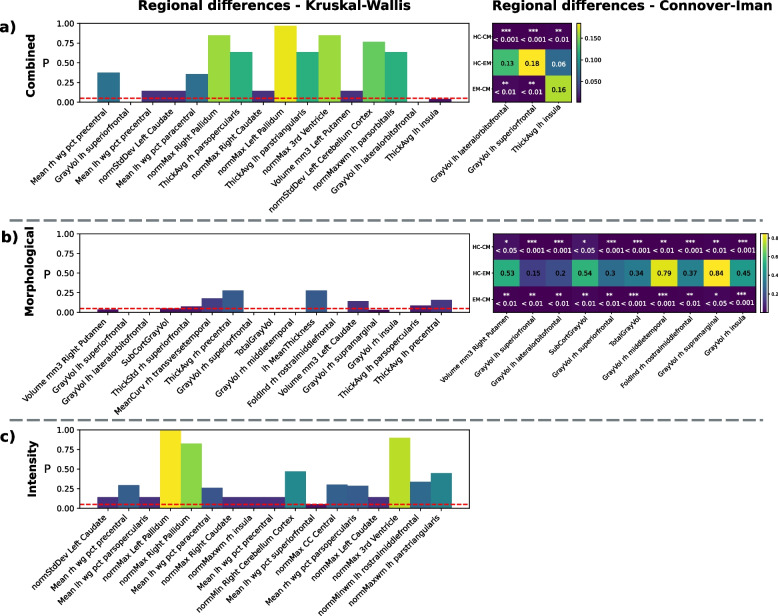
*P*-values derived from the Kruskal-Wallis test and the post-hoc Connover-Iman test for each of the most significant characteristics chosen for each regressor. Features are ranked from highest importance in the HC group to lower, left-right. **a** A total of 16 unique features were selected for the combined regressor, from which 3 demonstrated significant differences in the pairwise comparisons. **b** A total of 17 unique features were chosen for the regressor trained on the *Morphological Feature Set*. Up to 10 features demonstrated significant differences in importance between groups. **c** shows the 17 most significant characteristics for the regressor trained on the *Intensity Feature Set*. No significant differences were found during the Kruskal-Wallis test

Following the SHAP procedure described in Materials and methods, 16 features from the regressor trained on the *Combined Feature Set* were selected. Among them, SHAP values differed significantly between CM patients and HC for the left hemisphere lateral orbitofrontal cortex gray matter volume, left hemisphere superior frontal gyrus gray matter volume and the left hemisphere Insula average thickness (*p* < 0.001 for all cases). For the first two features, the left hemisphere lateral orbitofrontal cortex gray matter volume and the left hemisphere superior frontal gyrus gray matter volume, significant differences were also found in the SHAP values between EM and CM (*p* < 0.01 in both cases). No other significant differences were found for the remaining characteristics among the studied groups. These results are graphically depicted in Fig. 4.

Regarding the regressor trained on the *Morphological Feature Set*, 17 features were selected. Among them, nine features were significantly different between HC and CM, and ten, (the previous one plus one more) were significantly different for the EM-CM comparison. These are: the volume of the right Putamen (HC-CM *p* < 0.05, EM-CM *p* < 0.01), the volume of the superior frontal gyrus of the left hemisphere (HC-CM *p* < 0.001, EM-CM *p* < 0.01), the volume of the lateral orbitofrontal cortex of the left hemisphere (HC-CM *p* < 0.001, EM-CM < 0.01), the volume of the subcortical gray matter (EM-CM *p* < 0.01), the volume of the superior frontal gyrus of the right hemisphere (HC-CM *p* < 0.001, EM-CM *p* < 0.01), the total gray matter volume (HC-CM *p* < 0.001, EM-CM *p* < 0.001), the volume of the middle temporal gyrus of the right hemisphere (HC-CM *p* < 0.01, EM-CM *p* < 0.001), the folding index of the rostral middle frontal gyrus of the right hemisphere (HC-CM *p* < 0.001, EM-CM *p* < 0.01), the volume of the supramarginal gyrus of the right hemisphere (HC-CM *p* < 0.01, EM-CM *p* < 0.05) and the volume of the insula of the right hemisphere (HC-CM *p* < 0.001, EM-CM *p* < 0.001). These features and *P* values are also shown in Fig. 4.

Finally, no features from the regressor trained on the *Intensity Feature Set* (among the 17 that were selected) showed significant differences after the Kruskal-Wallis test. Feature importance for each ensemble studied are depicted Supplementary Figs. 4, 5, and 6.

### Relation between Brain Age Gap, imaging features and clinical characteristics

Employing the regressor trained on the *Combined Feature Set*, and both considering EM and CM separately or together, we found no significant association between the Brain Age Gap and headache frequency (CM - *p* = 0.89, EM - *p* = 0.72, both - *p* = 0.30), migraine frequency (CM - *p* = 0.71, EM - *p* = 0.62, both - *p* = 0.62), migraine duration in years (CM - *p* = 0.52, EM - *p* = 0.52, both - *p* = 0.52) or chronic migraine duration (*p* = 0.32). No significant associations were found either when considering the regressors trained on the *Morphological Feature Set* or the *Intensity Feature Set*. Results are shown in Supplementary Figs. 7, 8 and 9. Additionally, we analyzed the Pearson’s correlation between key important features and various clinical traits. To do this, we combined the features selected for the three predictive models and then identified unique features, totaling 40 in all. We found no statistically significant correlations. Results for this experiment are shown in Supplementary Fig. 10.

The mediation analysis results showed that the segmented brain volume mediates the relationship between headache frequency and Brain Age Gap when taking both groups as a whole. Detailed results can be seen in Supplementary Fig. 11.

## Discussion

In this study, we first applied the Brain Age paradigm to migraine patients using a machine learning model trained on brain T1w MRI data. Several important findings emerged from this investigation: First, CM patients had a statistically significant increase in predicted Brain Age compared to HC, whereas EM patients showed a nonsignificant increase. Second, different behaviors seem to arise when separately considering morphological and intensity-based features. Third, no associations were found between clinical characteristics such as headache frequency or disease duration and the Brain Age Gap or the imaging features that drive its prediction.

In the existing body of literature pertaining to the prediction of Brain Age, multiple methodologies have been employed to construct machine learning models. These methodologies range from feature-based approaches to advanced Deep Learning techniques [12, 19, 49]. Although Deep Learning techniques have demonstrated superior accuracy compared to feature-based methods - achieving an MAE as low as 2 to 3 years [50] as opposed to feature-based methods which infrequently reach an MAE below 4 years for the same age range and usually remain between an MAE of 5 to 6 years [51] - they are constrained by their ’black box’ nature and requirements for large data sets. Given that our study prioritized interpretability, we opted for a feature-based approach. We conducted a comparative analysis between three commonly employed machine learning algorithms: SVR, RF, and MLP. The MLP algorithm was selected for its superior performance metrics. During the validation phase, the MLP outperformed both the SVR and RF, achieving an MAE of 5.79 and a Pearson’s correlation coefficient of r=0.87. These results are consistent with similar approaches cited in the existing literature [51].

The Brain Age prediction model that was developed was later evaluated in three different contexts: first, using data from the Model Creation Dataset (a 10-fold cross-validation scheme was employed here). Second, data from the NKI-RS cohort was employed as an external validation dataset to ensure its generalization capabilities. Finally, the Brain Age model was evaluated on healthy subjects from the *Application Dataset*, in order to assess the accuracy of the model on the dataset that is relevant for our research question. Our results illustrate the model’s robust generalization capabilities as well as the efficacy of the feature harmonization method employed.

Combining different types of features, as we did with the *Combined Feature Set*, is a prevalent technique in computer vision [52–54]. The combination of these features is typically accompanied by an enhancement in the performance of the employed machine learning models [55], as is the case with our study. This is typically explained by the lack of correlation between distinct feature spaces. The separate results obtained for the Brain Age models trained using the *Morphological Feature Set* and the *Intensity Feature Set* allow us to gain more insight into the behavior of CM and EM patients since they offer complementary viewpoints of the nature of the Brain Age Gap in migraine. When employing the *Morphological Feature Set*, there is virtually no difference between HC and EM, whereas CM shows an increased Brain Age Gap that appears to vanish for older ages (see Fig. 3 (b) II). Although further investigation is required to corroborate this, it suggests that morphological changes in the brain that are associated with CM are more prominent at younger ages, but aging then absorbs these alterations. Conversely, the behavior of the Brain Age Gap when using the *Intensity Feature Set* seems more stable across ages (see Fig. 3 (c) II).

The interpretation of the Brain Age predicting model through SHAP allows us to better understand which brain imaging features mostly drive the Brain Age prediction, and which are responsible for the differences found. For the regressor trained on the *Combined Feature Set*, we identified a total of 16 features that highly influenced the prediction of the regressor across the studied groups. These characteristics primarily pertain to the frontal cortex (8), which is expected since features related to the frontal and temporal cortices are common in Brain Age models due to their generalized thinning as part of the normal process of aging [56]. Features related to the size of the ventricles are also common in Brain Age models, but not so much in our case (only two features). This is probably related to the fact that the increase in the volume of the ventricles is more pronounced after the sixth decade of life [57], while the subjects employed in our study ranged between 18 and 60 years of age. Ten out of the 16 most significant features were intensity-based, likely suggesting changes in the tissue microstructure [58].

The SHAP values of three of the brain imaging features found to be most relevant for the Brain Age estimation showed significant differences between CM patients and HC for the regressor trained on the *Combined Feature Set*. Additionally, regarding the regressor trained on the *Morphological Feature Set*, significant differences between CM and HC were found in the SHAP values of nine additional features (and nine more features in the comparison between EM and HC). Brain regions related to the identified features have been shown to differ morphologically [6], connectivity-wise [3, 59, 60], or both between HC and CM patients and between HC and EM patients. These regions are involved in complex cognitive functions such as information integration or working memory [61, 62] and their alteration may be related to the cognitive changes and other alterations associated with migraine [63, 64].

The reasons why migraine is associated with an increase in Brain Age can only be speculated at this point. Indeed, structural changes in the brain of migraine patients have been found from T1w MRI data [6], and some hypotheses have arisen such as the loss of cortical volume due to the damage induced by repetitive migraine attacks. The Brain Age paradigm condenses the changes that are associated with the aging of a healthy brain into one single number, the predicted Brain Age, and thus the same mechanisms can be hypothesized. This is in fact what our mediation analysis suggests. It seems that an increase in headache frequency is related to a decrease in brain volume which in turn might be related to an increased Brain Age Gap.

We found no correlations between the Brain Age Gap in migraine patients and clinical characteristics such as the frequency of headaches or migraine attacks or the duration of the disease. If the increased Brain Age Gap found in (chronic) migraine patients is a result of the damage produced by migraine attacks, one should expect a positive correlation between the Brain Age Gap and those clinical characteristics. Some other studies have also reported no correlation between the Brain Age Gap and illness duration for some pathologies [65], possibly implying that the alterations that cause an increase in the Brain Age primarily occur in the first stages of the disease. As migraine often ameliorates with age [66], the most severe impacts may occur early in the disease’s course, as suggested by our data. Alternatively, it is possible that the Brain Age Gap found in migraine patients does not actually reflect the effects of this condition, but rather a predisposition to suffer migraine that exists even before migraine appears. Unfortunately, the cross-sectional nature of our study prevents us from elucidating this question, and longitudinal analyses are needed to further investigate this issue. It is also important to notice that frequency has already been accounted for in the categorization of CM versus EM, which could conceal this correlation.

The interaction between migraine and brain aging has been studied before, although not from the perspective of the Brain Age paradigm. Chong et al. [10] examined if aging affects cortical thickness differently in patients with migraine compared to age-matched HC, potentially exacerbating cortical thinning in patients with migraine. For EM patients, the study found that patients with migraine experienced age-related thinning in regions that do not thin in HC. This suggests that migraine may be linked to atypical cortical aging. Lisicki et al., on the other hand, [11], employed FGD-PET to investigate possible specific age-related metabolic changes in the brain. They found that for EM patients advancing age was positively correlated to increased metabolism in the brainstem, hippocampus, fusiform gyrus and parahippocampus, regardless of the frequency of migraine or the duration of the disease. Taken together, these results are coherent with our study, both with regard to the existence of changes in the brain that are related to brain aging and with regard to the lack of association of some of these changes with disease duration.

It is also worth discussing our findings in the context of the broader spectrum of pain-related conditions. The existing literature presents conflicting results concerning Brain Age alterations in the context of chronic pain [20, 21]. Cruz-Almeida et al. endorse the view that there is an increased Brain Age Gap, whereas Soros et al. provide results that contradict this perspective. A subsequent study aimed to clarify this issue by examining three distinct chronic pain conditions, namely trigeminal neuralgia, osteoarthritis, and chronic back pain. The study found an increased Brain Age Gap in the first two conditions when comparing patients to age- and sex-matched controls, yet no such disparity was identified in the case of chronic back pain [65]. Further research is needed to elucidate whether different chronic-pain conditions share a common relationship with Brain Age increases, and where migraine lies within this context.

This research comes with several limitations. First, the cross-sectional design of the study limits our ability to establish causal relationships. This warrants a cautious interpretation of the findings and highlights the need for future longitudinal research to better understand the factors influencing the Brain Age Gap. Furthermore, additional phenotypic variables, such as cardiovascular risk metrics or cognitive performance scores, would have been helpful to clarify possible causal relationships mediated by the Brain Age Gap.

Secondly, our Brain Age model only employs T1w MRI data, though the inclusion of other imaging modalities such as diffusion MRI or fMRI has shown to improve the accuracy of Brain Age predictions [67]. However, incorporating other modalities would have led to the creation of distinct Brain Age models for each modality, since many of the datasets used for training do not include other modalities, which in turn would complicate the interpretation of the results. Alternatively, a much smaller sample size could have been used for training, which would have resulted in a less accurate model. Because of all these reasons, we chose to limit ourselves to T1w scans, although future work will need to include additional modalities such as diffusion MRI, as discussed earlier, or T2-weighted images, given their sensitivity to white matter hyperintensities, a well-known migraine characteristic associated with aging [68].

Third, high-frequency EM patients (10 to 14 headaches per month) were not included in the study. This decision was made to prevent potentially misclassified patients from skewing the results of the analysis since according to the literature [69] biologically they may resemble CM patients or they may even fulfill CM criteria part of the year.

Finally, given the moderate size of our sample and the much higher prevalence of migraine among women, few male subjects could be included, which prevented us from drawing any conclusions about the possible distinct behaviour of Brain Age in migraine between men and women.

## Conclusion

In this study, we analyzed migraine using the Brain Age framework, which consists of training a machine learning model to predict age from MRI scans and later applying the resulting model to a cohort of interest. We found that CM patients exhibit an increased Brain Age Gap (i.e., the difference between the predicted age and the chronological age) compared to HC. A milder Brain Age Gap was found for EM patients, although differences did not reach statistical significance.

Further analysis of the Brain Age model indicated that imaging features that have previously been associated with changes in migraine were among the main drivers of the differences in the predicted age. Also, a separate analysis using only morphological or intensity-based features revealed different patterns, which could represent distinct processes within the alterations that are associated with the migraine brain.

In conclusion, the Brain Age paradigm has shown to be a promising viewpoint for the study of migraine, and future work will be needed to corroborate these findings.

### Supplementary Information

**Additional file 1.**

**Additional file 2.**

## Data Availability

The public datasets used during the current study are available either online or after reasonable request. The institutional dataset used is also available author upon reasonable request to the corresponding author. The code used for modeling and data analysis is accessible at https://github.com/rafaloz/MigraineBA/.

## Abbreviations

ANCOVA: Analysis of covariance
ANOVA: Analysis of variance
brain-PAD: brain-predicted age difference
CM: Chronic migraine
CoRR: Consortium for Reliability and Reproducibility
DLBS: Dallas Lifespan Brain Study
EM: Episodic migraine
eTIV: Estimated total intracranial volume
FGD-PET: fluorodeoxyglucose positron emission tomography
fMRI: functional Magnetic Resonance Imaging
HC: Healthy controls
ICHD-3: International Classification of Headache Disorders
IXI: Information eXtraction from Images dataset
MAE: Mean absolute error
MLP: Multilayer perception
NeuroCog: Nerocognitive Aging release
RF: Random forest
SALD: Southwest University Adult Lifespan Dataset
SHAP: Shaply additive explanations
SVR: Support vector regressor
TE: Echo time
TFE: Turbo Field Echo
TR: Repetition time
T1w: T1-weighted

## References

[1] Arnold M (2018). Headache classification committee of the international headache society (IHS) the international classification of headache disorders. Cephalalgia.

[2] Ferrari MD, Goadsby PJ, Burstein R, Kurth T, Ayata C, Charles A, Ashina M, van den Maagdenberg AM, Dodick DW (2022) Migraine. Nat Rev Dis Prim 8(1). 10.1038/s41572-021-00328-4 10.1038/s41572-021-00328-4 35027572

[3] Jia Z, Yu S (2017). Grey matter alterations in migraine: A systematic review and meta-analysis. Neuroimage Clin.

[4] Kattem-Husøy A, Eikenes L, Håberg AK, Hagen K, Stovner LJ (2019). Diffusion tensor imaging in middle-aged headache sufferers in the general population: a cross-sectional population-based imaging study in the Nord-Trøndelag health study (HUNT-MRI). J Headache Pain.

[5] Schwedt TJ, Chiang CC, Chong CD, Dodick DW (2015). Functional MRI of migraine. Lancet Neurol.

[6] Planchuelo-Gómez Á, García-Azorín D, Guerrero ÁL, Rodríguez M, Aja-Fernández S, de Luis-García R (2020). Gray matter structural alterations in chronic and episodic migraine: a morphometric magnetic resonance imaging study. Pain Med.

[7] Planchuelo-Gómez Á, García-Azorín D, Guerrero ÁL, Aja-Fernández S, Rodríguez M, de Luis-García R (2020). White matter changes in chronic and episodic migraine: a diffusion tensor imaging study. J Headache Pain.

[8] Lee MJ, By Park, Cho S, Kim ST, Park H, Chung CS (2019). Increased connectivity of pain matrix in chronic migraine: a resting-state functional MRI study. J Headache Pain.

[9] Bell T, Khaira A, Stokoe M, Webb M, Noel M, Amoozegar F, Harris AD (2021). Age-related differences in resting state functional connectivity in pediatric migraine. J Headache Pain.

[10] Chong CD, Dodick DW, Schlaggar BL, Schwedt TJ (2014). Atypical age-related cortical thinning in episodic migraine. Cephalalgia.

[11] Lisicki M, D’Ostilio K, Coppola G, Parisi V, de Noordhout AM, Magis D, Schoenen J, Scholtes F, Versijpt J (2019). Age related metabolic modifications in the migraine brain. Cephalalgia.

[12] Franke K, Ziegler G, Klöppel S, Gaser C, Initiative ADN (2010). Estimating the age of healthy subjects from T1-weighted MRI scans using kernel methods: exploring the influence of various parameters. Neuroimage.

[13] Beheshti I, Mishra S, Sone D, Khanna P, Matsuda H (2020). T1-weighted MRI-driven brain age estimation in Alzheimer’s disease and Parkinson’s disease. Aging Dis.

[14] Ballester PL, Romano MT, de Azevedo Cardoso T, Hassel S, Strother SC, Kennedy SH, Frey BN (2022). Brain age in mood and psychotic disorders: a systematic review and meta-analysis. Acta Psychiatr Scand.

[15] Franke K, Luders E, May A, Wilke M, Gaser C (2012). Brain maturation: predicting individual BrainAGE in children and adolescents using structural MRI. Neuroimage.

[16] Rogenmoser L, Kernbach J, Schlaug G, Gaser C (2018). Keeping brains young with making music. Brain Struct Function.

[17] Steffener J, Habeck C, O’Shea D, Razlighi Q, Bherer L, Stern Y (2016). Differences between chronological and brain age are related to education and self-reported physical activity. Neurobiol Aging.

[18] Luders E, Cherbuin N, Gaser C (2016). Estimating brain age using high-resolution pattern recognition: Younger brains in long-term meditation practitioners. Neuroimage.

[19] Cole JH, Ritchie SJ, Bastin ME, Hernández V, Muñoz Maniega S, Royle N, Corley J, Pattie A, Harris SE, Zhang Q (2018). Brain age predicts mortality. Mol Psychiatry.

[20] Cruz-Almeida Y, Fillingim RB, Riley JL, Woods AJ, Porges E, Cohen R, Cole J (2019). Chronic pain is associated with a brain aging biomarker in community-dwelling older adults. Pain.

[21] Sörös P, Bantel C (2020). Chronic noncancer pain is not associated with accelerated brain aging as assessed by structural magnetic resonance imaging in patients treated in specialized outpatient clinics. Pain.

[22] Johnson AJ, Cole J, Fillingim RB, Cruz-Almeida Y (2022). Persistent Non-pharmacological Pain Management and Brain-Predicted Age Differences in Middle-Aged and Older Adults With Chronic Knee Pain. Front Pain Res.

[23] Yu GZ, Ly M, Karim HT, Muppidi N, Aizenstein HJ, Ibinson JW (2022). Accelerated brain aging in chronic low back pain. Brain Res.

[24] The Aging Mind Lab, University of Texas (2023) Dallas lifespan Brain Study. https://fcon_1000.projects.nitrc.org/indi/retro/dlbs.html. Accessed 20 Jan 2023

[25] Zuo XN, Anderson JS, Bellec P, Birn RM, Biswal BB, Blautzik J, Breitner J, Buckner RL, Calhoun VD, Castellanos FX (2014). An open science resource for establishing reliability and reproducibility in functional connectomics. Sci Data.

[26] Spreng RN, Setton R, Alter U, Cassidy BN, Darboh B, DuPre E, Kantarovich K, Lockrow AW, Mwilambwe-Tshilobo L, Luh WM (2022). Neurocognitive aging data release with behavioral, structural and multi-echo functional MRI measures. Sci Data.

[27] Marcus DS, Wang TH, Parker J, Csernansky JG, Morris JC, Buckner RL (2007). Open Access Series of Imaging Studies (OASIS): cross-sectional MRI data in young, middle aged, nondemented, and demented older adults. J Cogn Neurosci.

[28] Wei D, Zhuang K, Ai L, Chen Q, Yang W, Liu W, Wang K, Sun J, Qiu J (2018). Structural and functional brain scans from the cross-sectional Southwest University adult lifespan dataset. Sci Data.

[29] Biomedical Image Analysis Group, Imperial College London (2023) IXI dataset portal. https://brain-development.org/. Accessed 19 Jan 2023

[30] Taylor JR, Williams N, Cusack R, Auer T, Shafto MA, Dixon M, Tyler LK, Henson RN (2017). The Cambridge Centre for Ageing and Neuroscience (Cam-CAN) data repository: Structural and functional MRI, MEG, and cognitive data from a cross-sectional adult lifespan sample. Neuroimage.

[31] Shafto MA, Tyler LK, Dixon M, Taylor JR, Rowe JB, Cusack R, Calder AJ, Marslen-Wilson WD, Duncan J, Dalgleish T (2014). The Cambridge Centre for Ageing and Neuroscience (Cam-CAN) study protocol: a cross-sectional, lifespan, multidisciplinary examination of healthy cognitive ageing. BMC Neurol.

[32] Henschel L, Conjeti S, Estrada S, Diers K, Fischl B, Reuter M (2020). Fastsurfer-a fast and accurate deep learning based neuroimaging pipeline. NeuroImage.

[33] Klein A, Tourville J (2012). 101 labeled brain images and a consistent human cortical labeling protocol. Front Neurosci.

[34] Desikan RS, Ségonne F, Fischl B, Quinn BT, Dickerson BC, Blacker D, Buckner RL, Dale AM, Maguire RP, Hyman BT (2006). An automated labeling system for subdividing the human cerebral cortex on MRI scans into gyral based regions of interest. Neuroimage.

[35] Pomponio R, Erus G, Habes M, Doshi J, Srinivasan D, Mamourian E, Bashyam V, Nasrallah IM, Satterthwaite TD, Fan Y (2020). Harmonization of large MRI datasets for the analysis of brain imaging patterns throughout the lifespan. NeuroImage.

[36] Johnson WE, Li C, Rabinovic A (2007) Adjusting batch effects in microarray expression data using empirical Bayes methods. Biostatistics 8(1):118–127. 10.1093/biostatistics/kxj037 10.1093/biostatistics/kxj037 16632515

[37] Maia Polo F, Vicente R (2022) Effective sample size, dimensionality, and generalization in covariate shift adaptation. Neural Comput Applic 1–13. 10.1007/s00521-021-06615-1

[38] Butler ER, Chen A, Ramadan R, Le TT, Ruparel K, Moore TM, Satterthwaite TD, Zhang F, Shou H, Gur RC et al (2021) Pitfalls in brain age analyses. Technical report, Wiley Online Library. 10.1002/hbm.25533 10.1002/hbm.25533 PMC8357007 34190372

[39] de Lange AMG, Anatürk M, Rokicki J, Han LK, Franke K, Alnæs D, Ebmeier KP, Draganski B, Kaufmann T, Westlye LT et al (2022) Mind the gap: Performance metric evaluation in brain-age prediction. Hum Brain Mapp. 10.1002/hbm.25837 10.1002/hbm.25837 PMC9188975 35312210

[40] Pedregosa F, Varoquaux G, Gramfort A, Michel V, Thirion B, Grisel O, Blondel M, Prettenhofer P, Weiss R, Dubourg V (2011). Scikit-learn: Machine learning in Python. J Mach Learn Res.

[41] Paszke A, Gross S, Massa F, Lerer A, Bradbury J, Chanan G, Chintala S et al (2019) Pytorch: An imperative style, high-performance deep learning library. Adv Neural Inf Process Syst 32. 10.48550/arXiv.1912.01703

[42] Nooner KB, Colcombe SJ, Tobe RH, Mennes M, Benedict MM, Moreno AL, Panek LJ, Brown S, Zavitz ST, Li Q (2012). The NKI-Rockland sample: a model for accelerating the pace of discovery science in psychiatry. Front Neurosci.

[43] Olesen J, Bes A, Kunkel R, Lance JW, Nappi G, Pfaffenrath V, Rose FC, Schoenberg BS, Soyka D, Tfelt-Hansen P (2013). The international classification of headache disorders, (beta version). Cephalalgia.

[44] Zigmond A, Snaith R (1983). The Hospital Anxiety and Depression Scale. Acta Psychiatr Scand.

[45] Lundberg SM, Lee SI (2017). A unified approach to interpreting model predictions. Adv Neural Inf Process Syst.

[46] Ballester PL, Suh JS, Ho NC, Liang L, Hassel S, Strother SC, Arnott SR, Minuzzi L, Sassi RB, Lam RW (2023). Gray matter volume drives the brain age gap in schizophrenia: a SHAP study. Schizophrenia.

[47] Tabachnick BG, Fidell LS (2013). Using multivariate statistics.

[48] Lee PL, Kuo CY, Wang PN, Chen LK, Lin CP, Chou KH, Chung CP (2022). Regional rather than global brain age mediates cognitive function in cerebral small vessel disease. Brain Commun.

[49] Peng H, Gong W, Beckmann CF, Vedaldi A, Smith SM (2021). Accurate brain age prediction with lightweight deep neural networks. Med Image Anal.

[50] Tanveer M, Ganaie M, Beheshti I, Goel T, Ahmad N, Lai KT, et al (2023) Deep learning for brain age estimation: A systematic review. Inf Fusion 196:130–143. 10.1016/j.inffus.2023.03.007

[51] Mishra S, Beheshti I, Khanna P (2023) A review of neuroimaging-driven brain age estimation for identification of brain disorders and health conditions. IEEE Rev Biomed Eng 16:371–385. 10.1109/RBME.2021.3107372 10.1109/RBME.2021.3107372 34428153

[52] Gupta N, Bhatele P, Khanna P (2019). Glioma detection on brain MRIs using texture and morphological features with ensemble learning. Biomed Signal Process Control.

[53] Gumiran CR, Fajardo AC, Medina RP, Dao MS, Aguinaldo BE (2022) Aedes Aegypti Egg Morphological Property and Attribute Determination Based on Computer Vision. In: 2022 7th International Conference on Signal and Image Processing (ICSIP). IEEE, pp 581–585. 10.1109/ICSIP55141.2022.9887255

[54] Yan PF, Yan L, Hu TT, Xiao DD, Zhang Z, Zhao HY, Feng J (2017). The potential value of preoperative MRI texture and shape analysis in grading meningiomas: a preliminary investigation. Transl Oncol.

[55] Rathore S, Niazi T, Iftikhar MA, Chaddad A (2020). Glioma grading via analysis of digital pathology images using machine learning. Cancers.

[56] MacDonald ME, Pike GB (2021). MRI of healthy brain aging: A review. NMR Biomed.

[57] Bethlehem RA, Seidlitz J, White SR, Vogel JW, Anderson KM, Adamson C, Adler S, Alexopoulos GS, Anagnostou E, Areces-Gonzalez A (2022). Brain charts for the human lifespan. Nature.

[58] Salat DH, Lee SY, Van der Kouwe A, Greve DN, Fischl B, Rosas HD (2009). Age-associated alterations in cortical gray and white matter signal intensity and gray to white matter contrast. Neuroimage.

[59] Planchuelo-Gomez A, Garcia-Azorin D, Guerrero AL, Aja-Fernandez S, Rodriguez M, de Luis-Garcia R (2020). Structural connectivity alterations in chronic and episodic migraine: A diffusion magnetic resonance imaging connectomics study. Cephalalgia.

[60] Planchuelo-Gómez Á, García-Azorín D, Guerrero AL, Aja-Fernández S, Rodríguez M, de Luis-García R (2021). Multimodal fusion analysis of structural connectivity and gray matter morphology in migraine. Hum Brain Mapp.

[61] Boisgueheneuc Fd, Levy R, Volle E, Seassau M, Duffau H, Kinkingnehun S, Samson Y, Zhang S, Dubois B (2006). Functions of the left superior frontal gyrus in humans: a lesion study. Brain.

[62] Nogueira R, Abolafia JM, Drugowitsch J, Balaguer-Ballester E, Sanchez-Vives MV, Moreno-Bote R (2017). Lateral orbitofrontal cortex anticipates choices and integrates prior with current information. Nature Communications.

[63] Latysheva N, Filatova E, Osipova D, Danilov AB (2020). Cognitive impairment in chronic migraine: a cross-sectional study in a clinic-based sample. Arq Neuro-Psiquiatr.

[64] Luedtke K, Starke W, May A (2018). Musculoskeletal dysfunction in migraine patients. Cephalalgia.

[65] Hung PSP, Zhang JY, Noorani A, Walker MR, Huang M, Zhang JW, Laperriere N, Rudzicz F, Hodaie M (2022). Differential expression of a brain aging biomarker across discrete chronic pain disorders. Pain.

[66] Kelman L (2006). Migraine changes with age: Impact on migraine classification. Headache J Head Face Pain.

[67] Cole JH (2020). Multimodality neuroimaging brain-age in UK biobank: relationship to biomedical, lifestyle, and cognitive factors. Neurobiol Aging.

[68] Kruit MC, van Buchem MA, Launer LJ, Terwindt GM, Ferrari MD (2010). Migraine is associated with an increased risk of deep white matter lesions, subclinical posterior circulation infarcts and brain iron accumulation: the population-based MRI CAMERA study. Cephalalgia.

[69] Adams AM, Serrano D, Buse DC, Reed ML, Marske V, Fanning KM, Lipton RB (2015). The impact of chronic migraine: The Chronic Migraine Epidemiology and Outcomes (CaMEO) Study methods and baseline results. Cephalalgia.

