# Supplementary material for: Increased MRI-based Brain Age in chronic migraine patients
Source: J Headache Pain. 2023 Oct 6;24(1):133. doi: 10.1186/s10194-023-01670-6 (PMC10557155; doi:10.1186/s10194-023-01670-6)
Supplement: Supplementary file 1 — Additional file 1. [file 10194_2023_1670_MOESM1_ESM.pdf]

Additional file 1

Regions	Features	Total
**Subcortical regions (45)**	Volume; normalized intensity: mean, standard deviation, minimum, maximum and range	270
**Cortical regions left and right hemispheres (31x2)**	Area, volume, average thickness, thickness standard deviation, mean curvature, gaussian curvature, folding index, curvature index; White matter gray matter contrast: mean, standard deviation, minimum maximum and range	806
**White matter left and right hemispheres (32x2)**	Volume; normalized intensity: mean, standard deviation, minimum, maximum and range	384
**Whole brain features**		19
**Total**		**1479**

Supplementary Material Table 1: Features extracted by regions.

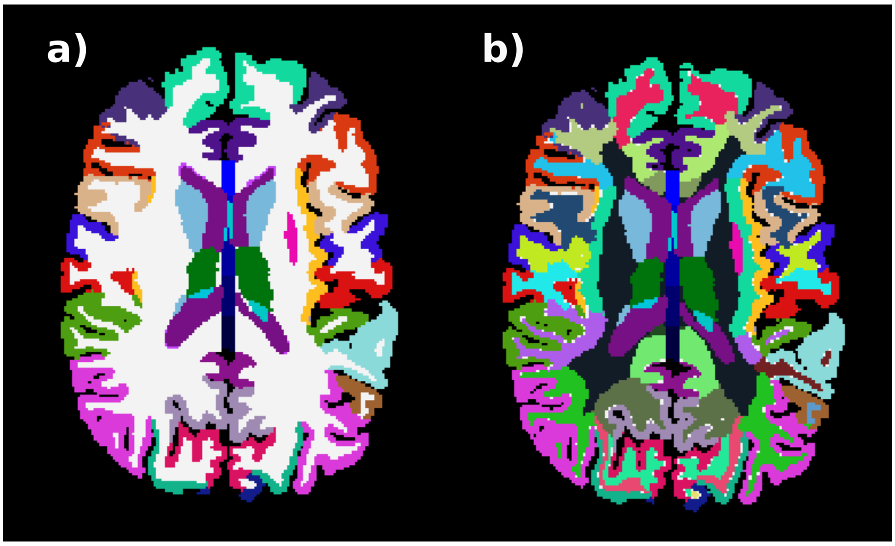

	Subcortical regions		
1	Left & Right Lateral-Ventricle	17	5th-Ventricle
2	Left & Right Inferior-Lateral-Ventricle	18	WM-hypointensities
3	Left & Right Cerebellum-White-Matter	19	non-WM-hypointensities
4	Left & Right Cerebellum-Cortex	20	Optic-Chiasm
5	Left & Right Thalamus-Proper	21	Corpus Callosum Posterior
6	Left & Right Caudate	22	Corpus Callosum Mid Posterior
7	Left & Right Putamen	23	Corpus Callosum Central
8	Left & Right Pallidum	24	Corpus Callosum MidAnterior
9	Left & Right Hippocampus	25	Corpus Callosum Anterior
10	Left & Right Amygdala	26	3rd-Ventricle
11	Left & Right Accumbens-area	27	4th-Ventricle
12	Left & Right VentralDC	28	Brain-Stem
13	Left & Right Vessel	29	CSF
14	Left & Right choroid-plexus		
15	Left & Right WM-hypointensities		
16	Left & Right non-WM-hypointensities		

	Cortical Regions		
1	Caudalanteriorcingulate	17	Parsorbitalis
2	Caudalmiddlefrontal	18	Parstriangularis
3	Cuneus	19	Pericalcarine
4	Entorhinal	20	Postcentral
5	Fusform	21	Posteriorcingulate
6	Inferiorparietal	22	Precentral
7	Inferiortemporal	23	Precuneus
8	Isthmuscingulate	24	Rostralanteriorcingulate
9	Lateraloccipital	25	Rostralmiddlefrontal
10	Lateralorbitofrontal	26	Superiorfrontal
11	Lingual	27	Superiorparietal
12	Medialorbitofrontal	28	Superiortemporal
13	Middletemporal	29	Supramarginal
14	Parahippocampal	30	Transversetemporal
15	Paracentral	31	Insula
16	Parsopercularis	32	UnsegmentedWhiteMatter

Supplementary Material Figure 1: Segmentation example from case 110033 of the CamCAN database. On the left a), the segmentation is shown without the white matter segmentation. Subcortical and cortical regions are divided. On the right b) the segmentation includes white matter segmentation.

**1**	Brain Segmentation Volume
**2**	Left hemisphere cortical gray matter volume
**3**	Right hemisphere cortical gray matter volume
**4**	Subcortical gray matter volume
**5**	Total gray matter volume
**6**	Supratentorial volume
**7**	Mask Volume
**8**	Number of defect holes in lh surfaces prior to fixing
**9**	Number of defect holes in rh surfaces prior to fixing
**10**	Estimated Total Intracranial Volume
**11**	Left Hemisphere White Surface Total Area
**12**	Right Hemisphere White Surface Total Area
**13**	Left Hemisphere Cortex Mean Thickness
**14**	Right Hemisphere Cortex Mean Thickness
**15**	Total cortical gray matter volume
**16**	Volume of ventricles and choroid plexus
**17**	Left hemisphere cerebral white matter volume
**18**	Right hemisphere cerebral white matter volume
**19**	Total cerebral white matter volume

Supplementary Material Table 2: Features extracted from the whole brain.

Regressors	Hyperparameters
SVR	kernel='linear', degree=3, gamma='scale', coef0=0.0, tol=0.001, C=1.0, epsilon=0.1, shrinking=True, cache_size=200
RF	n_estimators=100, criterion='squared_error', max_depth=None, bootstrap=True, min_samples_split=2, min_samples_leaf=1, min_weight_fraction_leaf=0.0, max_features=1.0, max_leaf_nodes=None, min_impurity_decrease=0.0, oob_score=False, ccp_alpha=0.0
MLP	epochs=500, lr=0.01, weigth_decay=0.01, validation_size=0.2, criterion=L1, optimizer=Adam, early_stopping=20 epochs

Supplementary Material Table 3: Hyperparameters of the regressors trained for the study.

Database	Escaner	Acquisition protocol
**The Open Access Series of Imaging Studies 1 (OASIS-1)**	1.5T Siemems Vision,<br>Washington University,<br>Saint Louis, Misuri, United States	MPRAGE; RT = 9.7 ms, ET = 4.0 ms, Flip Angle = 10°, IT = 20 ms,<br>DT = 200 ms, Orientation: Sagittal, thickness = 1.25 mm,<br>nº slices = 128, Resolution = 256 × 256 (1 × 1 mm)
**Information eXtraction from Images (IXI) initiative**	3T Philips Medical Systems Intera,<br>Hammersmith Hospital,<br>London, England, United Kingdom	RT = 9.6 ms, ET = 4.6 ms, Flip Angle = 8° Number of Phase Encoding Steps = 208,<br>Echo Train Length = 208, Reconstruction Diameter = 240.0, AcquisitionMatrix = 208 × 208,
	1.5T Philips Medical Systems Gyroscan Intera,<br>Guy's Hospital,<br>London, England, United Kingdom	RT = 9.8 ms, ET = 4.6 ms, Flip Angle = 8°, Number of Phase Encoding Steps = 192,<br>Echo Train Length = 0, Reconstruction Diameter = 240,
	Institute of Psychiatry,<br>London, England, United Kingdom	Not available
**NeuroCognitive Aging Data Release (NeuroCog)**	3T GE Discovery,<br>Cornell Magnetic Resonance Imaging Facility,<br>New York, New York, United States	MPRAGE; RT = 2530 ms, ET= 3.4 ms, Flip Angle = 7°,<br>voxel size = 1mm isotropic, acquisition time = 5m25s, 176 slices
	3T Siemens TimTrio,<br>York University Neuroimaging Center,<br>Toronto, Ontario, Canada	MPRAGE; RT = 1900 ms, ET = 2.52 ms, Flip Ange = 9°,<br>voxel size = 1mm isotropic, acquisition time = 4m26s; 192 slices
**Cambridge Center of Aging and Neuroscience (Cam-CAN)**	3 T Siemens TimTrio,<br>University of Cambridge,<br>Cambridge, England, United Kingdom	MPRAGE; RT = 2250 ms, ET = 2.99 ms, IT = 900ms, Flip Angle=9°, FOV=256 × 240 × 192mm,<br>resolution: 1mm isotropic; GRAPPA=2; acquisition time = 4mins 32s
**Southwest University Adult Lifespan Dataset (SALD)**	3T MRI Siemens TimTrio,<br>The Brain Imaging Center of Southwest University,<br>Beibei, Chongqing, China	MPRAGE; RT = 1.90 ms, ET=2.52 ms, TI=900 ms, Flip Angle = 90°,<br>resolution matrix = 256 × 256, slices = 176, thickness = 1,0 mm y voxel size = 1 × 1mm3
**Dallas Lifespan Brain Study (DLBS)**	3T Philips Achieva,<br>Park aging mind Laboratory,<br>Dallas, Texas, United States	MPRAGE; RT = 8.1 ms, ET = 3.7 ms, Flip Angle = 12°. Voxel size 1 × 1 × 1mm3,<br>slices = 160, matriz dimension 204 ×256 × 160
**Consortium for reliability and reproducibility (CoRR)**	35 different scaners from different institutions	Check parameters for each protocol at: https://www.nature.com/articles/sdata201449/tables/3

Supplementary Material Table 4: Acquisition parameters for each scanner employed in every database used to construct the Brain Age model.

		SVR		RF		MLP	
		**MAE**	**r**	**MAE**	**r**	**MAE**	**r**
**Combined Feature Set**	**20 features**	6.07 ± 0.29	0.82 ± 0.02	5.51 ± 0.25	0.84 ± 0.02	5.03 ± 0.29	0.86 ± 0.02
	**30 features**	5.94 ± 0.24	0.83 ± 0.02	5.54 ± 0.26	0.84 ± 0.02	4.92 ± 0.25	0.86 ± 0.02
	**40 features**	5.85 ± 0.22	0.83 ± 0.02	5.55 ± 0.27	0.84 ± 0.02	**4.90 ± 0.21**	**0.87 ± 0.01**
**Morphological Feature Set**	**20 features**	6.68 ± 0.43	0.78 ± 0.02	6.53 ± 0.54	0.76 ± 0.03	5.74 ± 0.47	0.80 ± 0.02
	**30 features**	6.52 ± 0.44	0.79 ± 0.02	6.54 ± 0.46	0.77 ± 0.03	5.66 ± 0.44	0.81 ± 0.02
	**40 features**	6.37 ± 0.39	0.80 ± 0.02	6.46 ± 0.42	0.77 ± 0.02	**5.57 ± 0.27**	**0.81 ± 0.02**
**Intensity Feature Set**	**20 features**	6.91 ± 0.41	0.75 ± 0.02	6.64 ± 0.40	0.75 ± 0.02	6.17 ± 0.40	0.77 ± 0.02
	**30 features**	6.87 ± 0.46	0.75 ± 0.02	6.67 ± 0.43	0.76 ± 0.02	6.13 ± 0.44	0.78 ± 0.03
	**40 features**	6.80 ± 0.38	0.76 ± 0.02	6.72 ± 0.41	0.75 ± 0.02	**6.06 ± 0.35**	**0.78 ± 0.02**

Supplementary Material Table 5: Validation results for the three regressors tested. Results are given as the average and the standard deviation of the values obtained from each fold of the 10-fold cross-validation scheme before age bias correction. The values in bold show the combination with the best result.

		F	P-val	np2
**ANCOVA HC-CM-EM**	**Brain Age Gap**	2.969	0.053	0.240
	**eTIV**	14.666	<0.001	0.057
	**Sex**	0.213	0.645	<0.001
**ANCOVA HC-EM**	**Brain Age Gap**	1.734	0.019	0.001
	**eTIV**	9.900	0.002	0.005
	**Sex**	<0.001	0.997	<0.001
**ANCOVA HC-CM**	**Brain Age Gap**	6.796	0.010	0.043
	**eTIV**	4.744	0.031	0.030
	**Sex**	0.428	0.514	0.003
**ANCOVA EM-CM**	**Brain Age Gap**	1.110	0.294	0.007
	**eTIV**	15.749	<0.001	0.089
	**Sex**	0.175	0.676	0.001

Supplementary Material Table 6: ANCOVA complete results for Brain Age Gap calculated for the combined regressor. Normality and equality of variances were tested before applying the ANCOVA. Sex and eTIV were included as covariates.

		F	P-val	np2
**ANCOVA HC-EM-CM**	**Brain Age Gap**	1.840	0.161	0.015
	**eTIV**	20.37	0.078	<0.001
	**Sex**	2.423	0.010	0.121
**ANCOVA HC-EM**	**Brain Age Gap**	0.102	0.750	0.001
	**eTIV**	11.49	<0.001	0.064
	**Sex**	3.878	0.051	0.022
**ANCOVA HC-CM**	**Brain Age Gap**	3.237	0.074	0.021
	**eTIV**	9.191	0.003	0.057
	**Sex**	0.659	0.418	0.004
**ANCOVA EM-CM**	**Brain Age Gap**	1.924	0.167	0.012
	**eTIV**	21.01	<0.001	0.115
	**Sex**	1.214	0.272	0.008

Supplementary Material Table 7: ANCOVA complete results for Brain Age Gap calculated for the intensity regressor. Normality and equality of variances were tested before applying the ANCOVA. Sex and eTIV were included as covariates.

		F	P-val	np2
**ANCOVA HC-EM-CM**	**Brain Age Gap**	2.156	0.118	0.018
	**eTIV**	0.999	0.319	0.004
	**Sex**	3.952	0.048	0.016
**ANCOVA HC-EM**	**Brain Age Gap**	1.802	0.181	0.011
	**eTIV**	0.074	0.786	<0.001
	**Sex**	2.794	0.096	0.016
**ANCOVA HC-CM**	**Brain Age Gap**	4.094	0.045	0.026
	**eTIV**	0.707	0.402	0.005
	**Sex**	1.844	0.176	0.012
**ANCOVA EM-CM**	**Brain Age Gap**	0.336	0.563	0.002
	**eTIV**	3.280	0.072	0.020
	**Sex**	2.515	0.115	0.015

Supplementary Material Table 8: ANCOVA results for Brain Age Gap calculated for the morphological regressor. Normality and equality of variances were tested before applying the ANCOVA. ETIV was included as a covariate.

		ANCOVA HC-EM	ANCOVA HC-CM	ANCOVA EM-CM
	**F-value**	2.387	5.581	0.440
**Combined Feature Set**	**Effect size (η_p^2)**	0.017	0.040	0.003
	**p-value**	0.125	0.020	0.508
	**F-value**	0.218	2.373	0.968
**Morphological Feature Set**	**Effect size (η_p^2)**	0.002	0.018	0.007
	**p-value**	0.641	0.126	0.327
	**F-value**	1.618	5.555	0.666
**Intensity Feature Set**	**Effect size (η_p^2)**	0.011	0.040	0.005
	**p-value**	0.205	0.020	0.416

Supplementary Material Table 9: ANCOVA results for Brain Age Gap calculated for the female subgroup. Normality and equality of variances were tested before applying the ANCOVA. Sex and eTIV were included as covariates.

		ANCOVA HC-EM	ANCOVA HC-CM	ANCOVA EM-CM
**Combined Feature Set**	**F-value**	0.950	6.789	0.509
	**Effect size (η_p^2)**	0.035	0.047	0.028
	**p-value**	0.339	0.388	0.485
**Morphological Feature Set**	**F-value**	0.638	0.746	1.239
	**Effect size (η_p^2)**	0.024	0.045	0.064
	**p-value**	0.432	0.400	0.280
**Intensity Feature Set**	**F-value**	2.171	0.114	1.088
	**Effect size (η_p^2)**	0.077	0.007	0.057
	**p-value**	0.153	0.740	0.311

Supplementary Material Table 10: ANCOVA results for Brain Age Gap calculated for the male subgroup. Normality and equality of variances were tested before applying the ANCOVA. Age and eTIV were included as covariates.

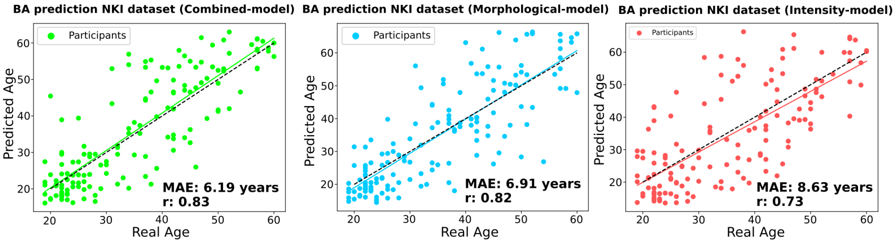

Supplementary Material Figure 2: Results of the Brain Age models on the external validation dataset (NKI-RS). The performance of the models is similar to that obtained on the healthy controls of the Application Dataset, thereby confirming the generalizability and reliability of the models.

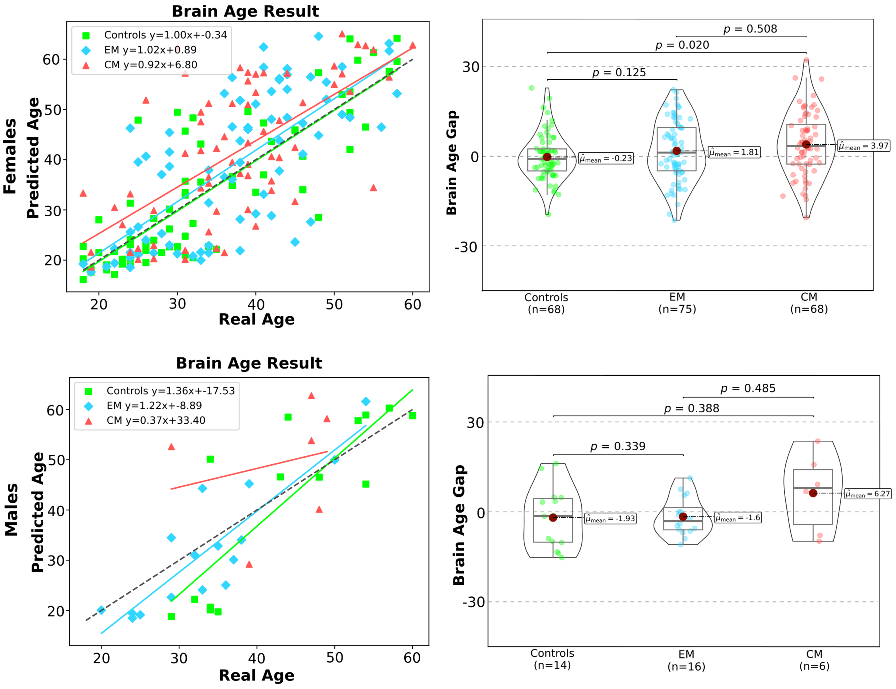

Supplementary Material Figure 3: The outcomes derived from the integrated regression model for each gender are presented. While the findings do not indicate any statistically significant differences, they do suggest that females are the predominant factor contributing to the disparity between HC and CM. It is important to interpret these results cautiously, given the limited sample size of the male group.

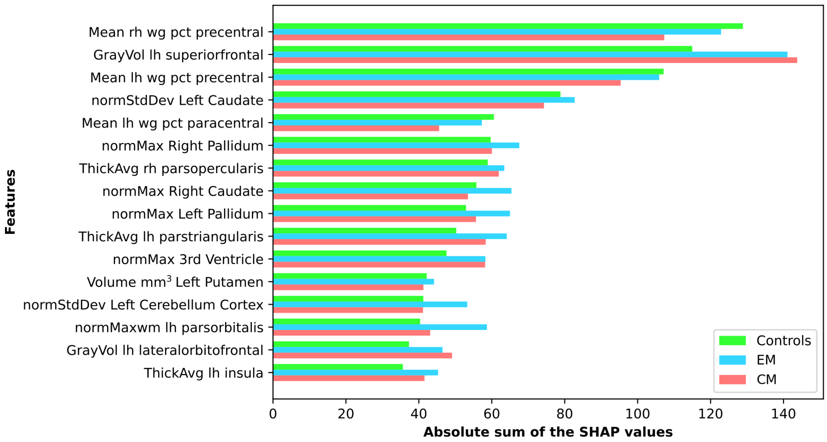

Supplementary Material Figure 4: The sum of the absolute SHAP values of each feature for each member of the investigated groups, calculated for the regressor trained on the *Combined Feature Set*. The order of features varies between groups, but the 16 designated features are shared by the groups' most pertinent features.

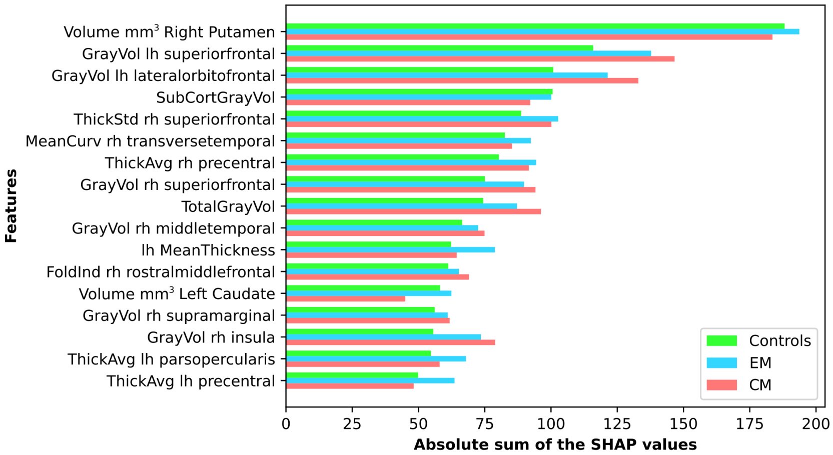

Supplementary Material Figure 5: The sum of the absolute SHAP values of each feature for each member of the investigated groups, calculated for the regressor trained on the *Morphological Feature Set*. The order of features varies between groups, but the 17 designated features are shared by the groups' most pertinent features.

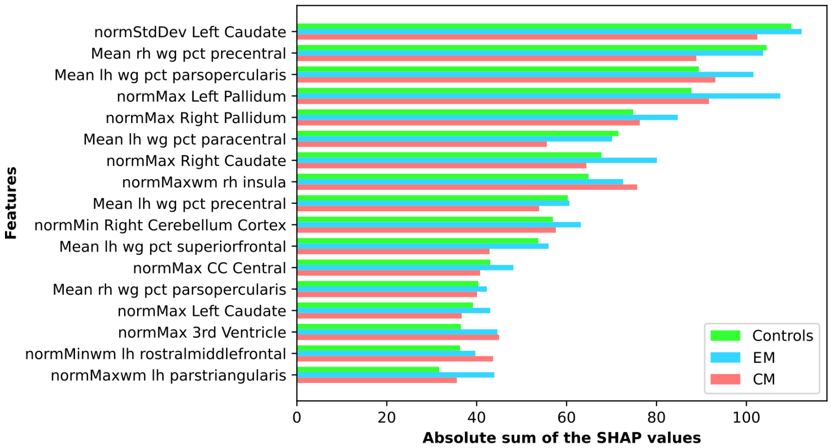

Supplementary Material Figure 6: The sum of the absolute SHAP values of each feature for each member of the investigated groups, calculated for the regressor trained on the *Intensity Feature Set*. The order of features varies between groups, but the 17 designated features are shared by the groups' most pertinent features.

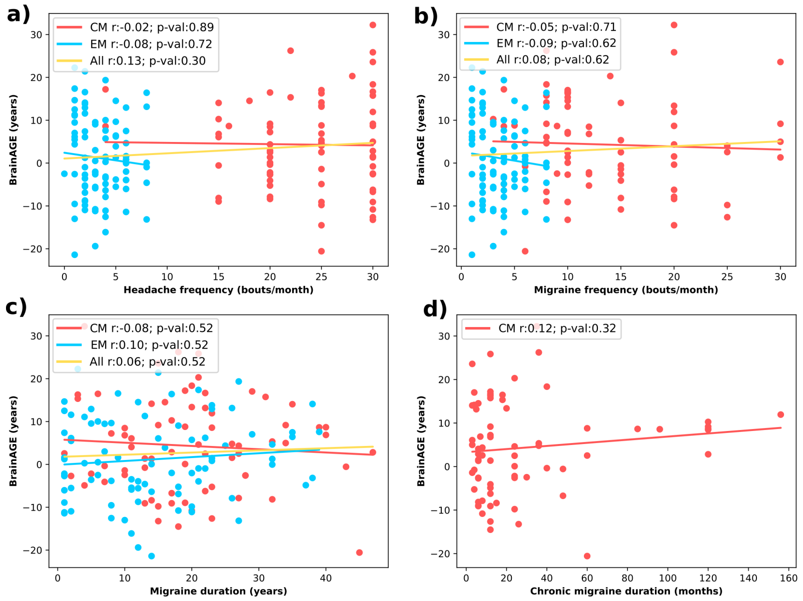

Supplementary Material Figure 7: No correlations were found between Brain Age Gap calculated with the regressor trained on the *Combined Feature Set* and the clinical variables studied, a) Brain Age Gap change along with headache frequency, b) Brain Age Gap change along with migraine frequency, c) Brain Age Gap change along with migraine duration, and d) Brain Age Gap change along with chronic migraine duration.

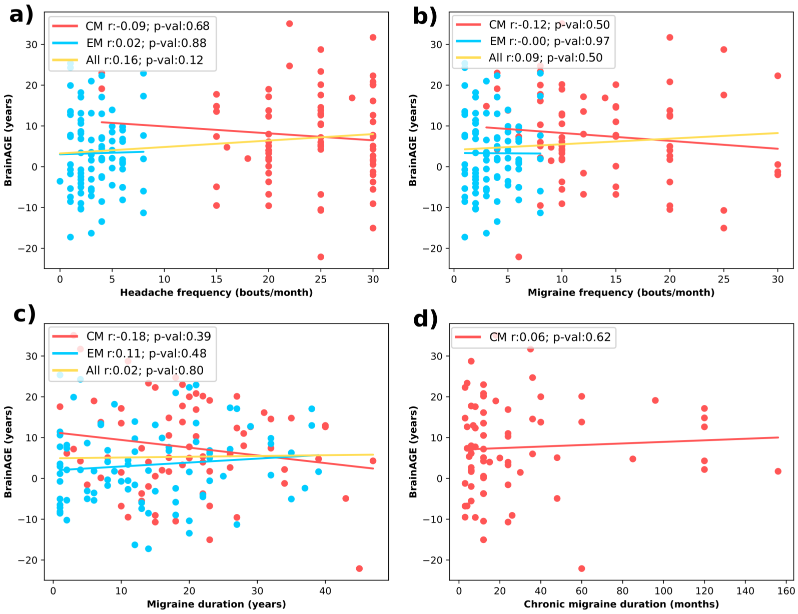

Supplementary Material Figure 8: No statistically significant correlation was found between clinical variables and Brain Age Gap when calculated with the regressor trained on the *Morphological Feature Set*, a) Brain Age Gap change along with headache frequency, b) Brain Age Gap change along with migraine frequency, c) Brain Age Gap change along with migraine duration, and d) Brain Age Gap change along with chronic migraine duration.

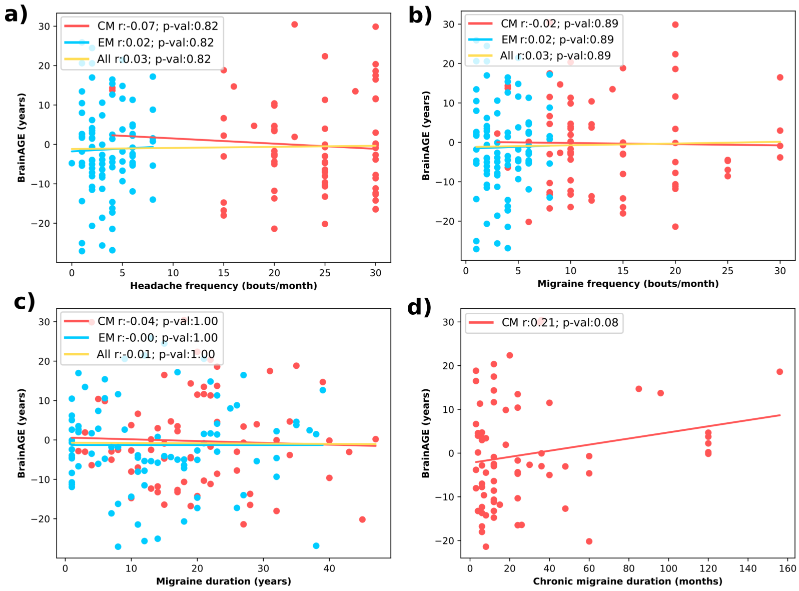

Supplementary Material Figure 9: No statistically significant correlation was found between the clinical variables and the Brain Age Gap when calculated with the regressor trained on the *Intensity Feature Set*, a) Brain Age Gap change along with headache frequency, b) Brain Age Gap change along with migraine frequency, c) Brain Age Gap change along with migraine duration, and d) Brain Age Gap change along with chronic migraine duration.

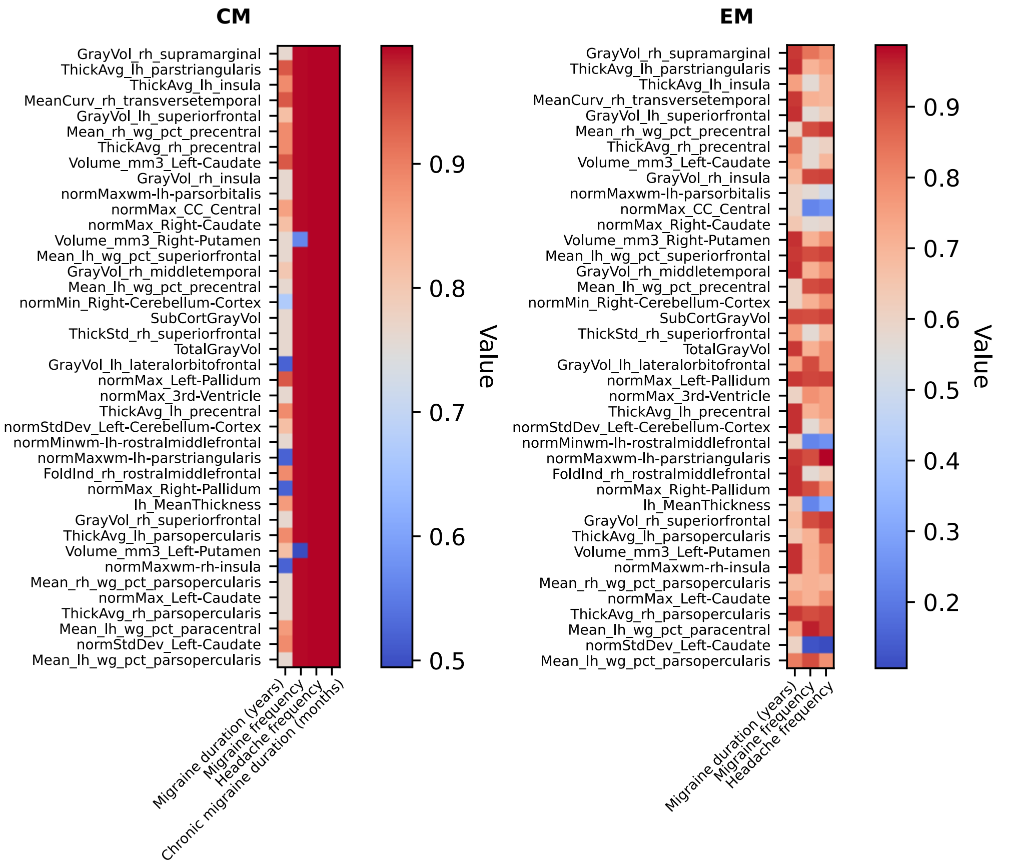

Supplementary Material Figure 10: No statistically significant correlations were found between the selected key features during the model interpretation and the clinical variables of the CM and EM patients.
